# *Dolichos lablab* L. extracts as pharmanutrient for stress-related mucosal disease in rat stomach

**DOI:** 10.3164/jcbn.20-11

**Published:** 2020-06-11

**Authors:** Jeong Min An, EunHye Kim, Ho Jae Lee, Min Hee Park, Dong Ju Son, Ki Baik Hahm

**Affiliations:** 1 CHA Cancer Preventive Research Center, CHA Bio Complex, CHA University, 59, Yatap-ro, Bundang-gu, Seongnam-si, Gyeonggi-do, 13496, Korea; 2 Gachon University Lee Gil Ya Cancer and Diabetes Institute, Incheon, 21565, Korea; 3 Division of Life and Pharmaceutical Sciences, Ewha Womans University, Seoul, 03760, Korea; 4 College of Pharmacy and Medical Research Center, Chungbuk National University, Cheongju, 28644, Korea; 5 Research Institute, Medpactor, 92, Myeongdal-ro, Seocho-gu, Seoul, 06668, Korea

**Keywords:** SRMD, *Dolichos lablab* L., cytoprotection, Nrf2, HO-1

## Abstract

Gastric stress-related mucosal disease (SRMD) presented from superficial gastritis to deep ulceration consequent to insufficient perfusion, ischemia, and oxidative stress. Though pharmacologic interventions to optimize tissue perfusion or to enhance defensive mechanism are essential, limited clinical outcome necessitates strong acid suppressors or natural agents. Under the hypothesis that *Dolichos lablab* L. (NKM 23-1) can enhance defense against SRMD, water immersion restraint stress (WIRS) were imposed to rats and additional groups pretreated with differing doses of NKM 23-1 were monitored. On gross and microscopic evaluation, they significantly rescued SRMD (*p*<0.01). The levels of inflammatory mediators such as IL-18, IL-1β, IL-8, iNOS, TNF-α, caspase-1, NOXs as well as MMPs accompanied with NF-κB p50 activation were all significantly increased in WIRS, but their levels were significantly decreased in Groups pretreated with NKM 23-1. WIRS significantly increased apoptosis, but significantly decreased with NKM 23-1 accompanied with significantly increased levels of cyclin D/E and HSP70/HSP27. Gastric mucin was significantly preserved in Groups pretreated with NKM 23-1, while depleted in WIRS, accompanied with increased expressions of Muc5A. Gastric levels of HO-1 and NQO1 were significantly increased in Group treated with NKM 23-1 with transcriptional activation of Nrf2. Conclusively, preemptive intake of NKM 23-1 significantly rescued SRMD.

## Introduction

Gastric stress-related mucosal disease (SRMD) is a common clinical finding to encompass the continuum from stress-related mucosal damage to stress ulceration in human as well as veterinary area, which is defined from superficial mucosal erosions to deeper ulcers, some presenting with overt gastrointestinal (GI) bleeding, causing hemodynamic instability and hypovolemic shock. Though its incidence is high in critically ill human patients admitted in intensive care unit (ICU), it develops in non-ICU patient.^(1–5)^ Though exact pathogenesis is not elucidated, multi-factorial and complex, yet, ischemia related to stress, some infection like *Helicobacter pylori*, decreased host defenses as seen in toxic drug like nonsteroidal anti-inflammatory drug, and systemic illness can lead to enormous gastric acid attack and deranged gastric defenses,^(2–5,6,7)^ after which therapy is aimed to eliminate causing etiologies, to enhance defense system, and to decreased gastric acids.^(7,8)^ Following ischemia/reperfusion after stress, proinflammatory mediators such as TNF-α, IL-1β, IL-8, iNOS caused leukocyte activation, endothelial cell swelling, and vascular leakage, finally presented with gastric erosions/ulcers.^(3,4,6)^

Since the final blow for gastric mucosal destruction is executed by gastric acids, acid prophylaxis is usually recommended to decrease gastric acidity using antacid, histamine receptor antagonist, and proton pump inhibitors including omeprazole and pantoprazole with alleviating conditions and economic impact.^(2,7)^ However, in spite of reduced major bleeding with acid suppressants, little or no effect of acid suppressive therapy was noted on mortality and patients in non-intensive care unit.^(8,9)^ As much as pharmacologic therapy with anti-secretary drugs, enhanced gastric defense system can be alternate or combinational enhancer to cover the limitation of acid suppressive therapy since pre-epithelial factors, an epithelial barrier, and subepithelial factors are also principally implicated in SRMD.^(10)^

White bean extract containing *Dolichos lablab* L. (NKM 23-1) enriched with two triterpene glycosides 1 and 2, had been reported to alleviate irritable bowel syndrome-like symptoms,^(11)^ to ameliorate obesity,^(12)^ and to prevent fatty liver disease.^(13)^ Upon studies including bioactive, biochemical, physicochemical, and functional properties of NKM 23-1, *Dolichos lablab* L., contains many health-promoting components such as fiber, proteins, minerals, and numerous phytochemicals like flavonoids, saponins, and polyphenols.^(14)^ Based on these characterizations, we have found the quite cytoprotective outcome of NKM 23-1 in HCl- or ethanol-stimulated gastric ulcer models.

In this study, under the hypothesis that nutritional intervention using white bean extract can afford protection against SRMD initiated with water immersion restraint stress (WIRS), we performed efficacy evaluation test with exploration of pharmacological mechanisms. Our study was intended for future implication of dietary intake of *Dolichos lablab* L. white bean extracts as secure preventive way against SRMD, especially in persons under stimulation of gastric irritation.

## Materials and Methods

### WIRS model to induce SRMD

A total of 110 rats (Sprague-Dawley; SD) were purchased from Charles River (Osaka, Japan) and kept in an animal facility. Animals were handled in an accredited animal facility in accordance with the AAALAC International Animal Care Policies of CHA Bio Complex (CHA University, Seongnam, Korea) after IRB approving (#2019-0301). The animals were deprived of food, but allowed free access to water 24 h before exposure of WIRS. Ten rats in each group were placed in strained cages and immersed in water (WIRS) for 6 h. Animals were killed immediately after the end of 6 h WIRS. Fifty rats were subjected to WIRS and gross lesion index were determined in 6 groups (*n* = 10/group). After this preliminary study, animals were repeatedly divided into four groups as follows: normal rats without any intervention except oral administration of normal saline, WIRS group as applying WIRS for 6 h, NKM 23-1 25 mg/kg group as NKM 23-1 25 mg/kg per oral (p.o.) pretreatment 8 h before applying WIRS, NKM 23-1 50 mg/kg group as NKM 23-1 50 mg/kg p.o. pretreatment 8 h before applying WIRS, and NKM 23-1 100 mg/kg group as NKM 23-1 100 mg/kg p.o. pretreatment 8 h before applying WIRS. Animals were killed with high dose intraperitoneal anesthesia (thiopental sodium, 50 mg/kg). The stomachs of rats were removed and opened along the greater curvature, and then washed with iced cold PBS solutions. The number and size of either erosions or ulcers were determined under the magnified photographs, after which half of each dissected. Stomach was spread onto a plastic sheet, fixed in 10% buffered formalin for 4 h, and prepared for paraffin tissue slides and the remaining half was kept in a liquid nitrogen tank for further molecular study. The mucosal homogenates were pooled together.

### Macroscopic and microscopic evaluation

The stomach isolated from the rat was placed in 10% buffered formalin and embedded in paraffin, and sections were cut. With the modification of the criteria of Yamamoto *et al.*,^(15)^ the bleeding index was evaluated on formalin-fixed sections and the bleedingrate (%) was calculated. Simply stated, index 0 means no bleeding at all, index 1 is mild bleeding showing the presence of small amounts of coagula in the stomach, index 2 is moderate bleeding showing intermediate state between 1 and 3 points, and index 3 is severe bleeding that contents of the stomach were filled with bleedings including coagula.

### Preparation of *Dolichos lablab* L. extract for NKM 23-1

Dried seeds of *Dolichos lablab* was purchased from Kwangmyungdang Medical Herbs Inc. (Ulsan, Korea) in November 2018. A voucher specimen has been deposited in the Life Science Research Center (NKM-DLL2018-11-01). Dried *D. lablab* (total 30 kg) was extracted with distilled water (30 L) for 3 h at 90–100°C. The crude extract solution was filtered, concentrated by boiling in open vessel at atmospheric pressure, and then pulverized by spray-drying, to yield 2.84 kg (dry weight) of NKM-23-001 (10.6% w/w yield). The NKM-23-01 was packed in vacuum-sealed aluminum foil pouch (100 g/pouch) and stored at room temperature until used.

### RT-PCR

Total RNA was extracted using an RNeasy Mini kit (Qiagen Korea, Seoul, Korea). Primers used for inflammatory cytokines and mediators were shown in Table 1. The amplifications were done in 50-ml reaction volumes containing 10× reaction buffer (Promega Korea, Seoul, Korea), 1.5 mM MgCl_2_, 200 mM deoxynucleotide triphosphates (dNTP), 1 mM of each primer, and 2.5 units of *Taq* DNA polymerase (Promega) using a Perkin-Elmer Gene Amp PCR System 2400. Each cycle consists of denaturation at 95°C for 1 min, annealing at 55°C for 45 s, and amplification at 72°C for 45 s.

### Western blotting

Gastric mucosa was homogenized in ice-cold 20 mM Tris-HCl buffer, pH 7.5, containing 2 mM ethylene diamine tetraacetic acid (EDTA), 0.5 mM ethylene glycol tetraacetic acid (EGTA), 300 mM sucrose, and 2 mM phenyl methyl sulfonyl fluoride with a tissue homogenator. Thirty µg of the protein was subjected to electrophoresis on an 8% sodium dodecyl sulfate-polyacrylamide gel electrophoresis (SDS-PAGE) gel and transferred onto polyvinylidene fluoride (PVDF) membrane using a semidry transfer system (Hoefer, Holliston, MA). Non-specific bindings were blocked by incubation with 5% non-fat dry milk. The membranes were incubated overnight at 4°C with a 1:500 dilution of each primary antibody in blocking solution, followed by incubation with 1:1,000 dilution of horseradish peroxidase (HRP)-conjugated secondary antibody. The immune-complexes were detected using an ECL detection kit (Amersham Biosciences Korea, Seoul, Korea) and auto-radiographed onto X-ray film. The gastric mucosal homogenates were pooled together in order to compare. The antibodies used for Western blot are as follow; β-actin, nitric oxide synthase (iNOS), cyclooxygenase (COX-2), phosphor-JNK (P-JNK), phosphor-ERK (P-ERK), cyclin E, CDKs, and cyclin D1 antibodies were purchased from Santa Cruz Biotechnology (Santa Cruz, CA), phosphorylated Inhibitor of Kappa B alpha (p-IκBα), phosphorylated signal transducer and activator of transcription 3 (p-STAT3), caspase-1, cyclin-dependent kinase 4 (CDK4) and cyclin-dependent kinase 2 (CDK2) were from Cell Signaling Technology (Beverly, MA). A heme oxygenase-1 (HO-1) antibody was obtained from Enzo Life Sciences (Farmingdale, NY)

### Terminal deoxynucleotidyl transferase-mediated nick end labeling

Apoptosis was visualized with terminal *TdT* FragEL DNA fragmentation detection kit (Oncogene Research Products, Cambridge, MA). To determine the apoptotic index (AI) in each group, we first scanned terminal deoxynucleotidyl transferase mediated - dUTP nick end labeling (TUNEL) - immunostained sections under ×100 magnification to locate the apoptotic hotspots. Then, AI under ×200 magnified field was scored by counting the number of TUNEL-positive cells. At least five hot spots in a section containing erosive or ulcerative lesions were randomly selected and average count was determined. Data were expressed as a mean percentage of total cell numbers.

### PAS staining

Neutral glycoproteins (mucin) contents were determined by periodic acid–Schiff (PAS) staining in the stomach tissues. In detail, for periodic acid and Schiff’s (PAS) staining, histochemical staining of glycoconjugates was carried out as per the method of Pandurangan *et al.*,^(16)^ using 2% PAS reagent in dark for 20 min.

### Statistical analysis

Results are expressed as the mean ± SD. The data were analyzed by one-way analysis of variance (ANOVA), and the statistical significance between groups was determined by Duncan’s multiple range test. Statistical significance was accepted with *p*<0.05.

## Results

### NKM 23-1 pretreatment ameliorated SRMD in rats

**Fig. 1:**
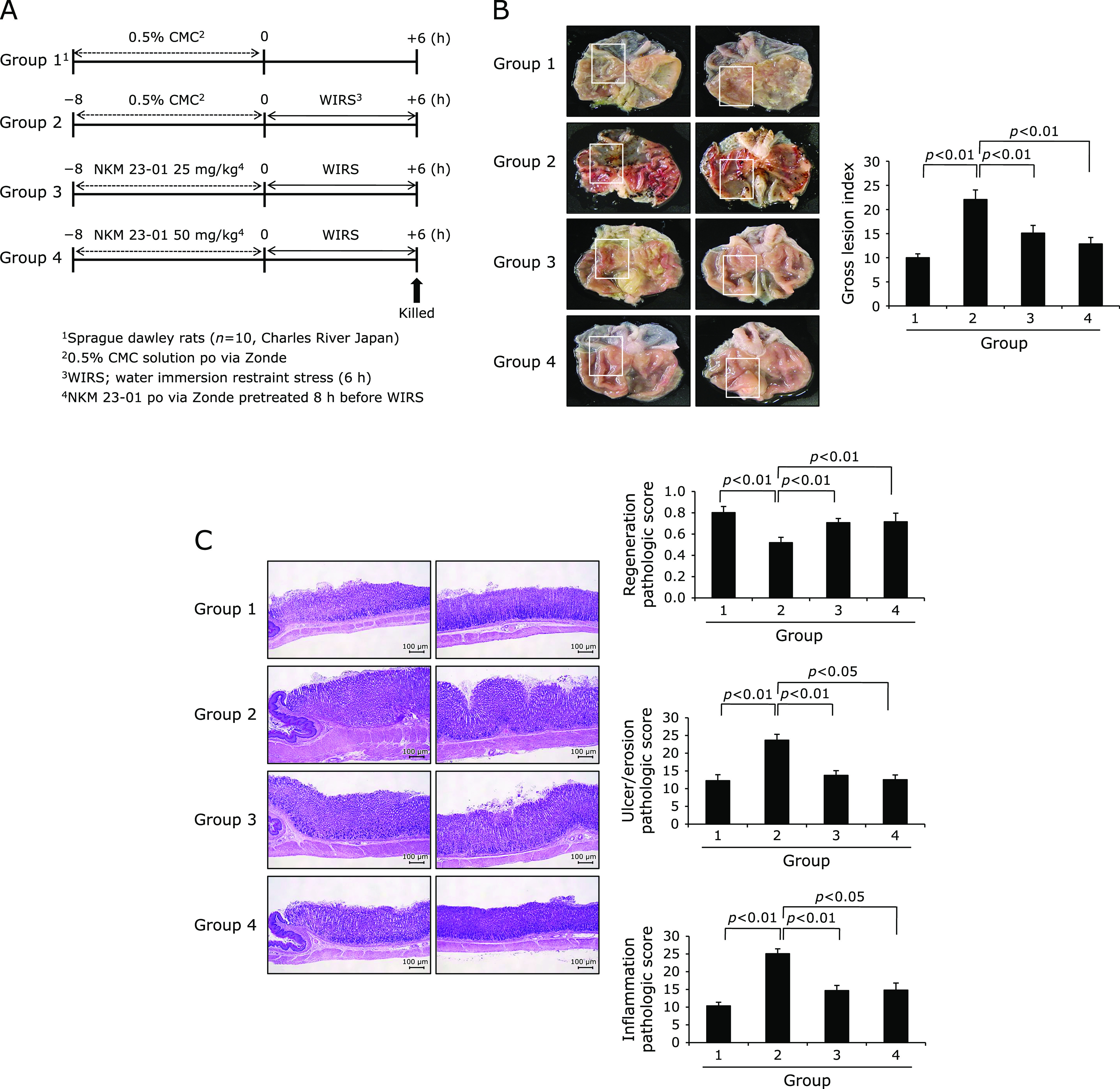
Preventive action of NKM 23-1 against WIRS-induced gastric mucosal damages. (A) Experimental protocol for WIRS-induced gastric mucosal damage. In order to document the preventive effects of NKM 23-1 extracts (extract of *Dolichoslablab* L.), NKM 23-1 was pretreated 8 h before imposing WIRS (6 h). Three different doses of NKM 23-1, 25, 50, and 100 mg/kg, were administered via zoned, respectively in SD rats (*n* = 10). (B, C) Gross and pathological photography. Two representational photosper group were displayed, ×40 magnification on pathology showing multiple ulcers/erosions on group 2. Lesion score of mean gross and pathology. Gross lesion scores consisted of hemorrhage, edema, and ulcer/erosion and pathological scores separately scored for inflammation, mucosal ulceration, and regenerative activities, respectively, ×40 magnification. Mean pathologies were evaluated in two points, border covering fore-stomach and stomach portion and glandular portion of stomach.

 As stress related mucosal damages (SRMD), we imposed water immersion restraint stress for 6 h. As result, significant gastric damages including linear hemorrhagic ulcers and scattered erosions with mucosal edema were induced in rat stomach (Fig. 1B). However, these gross lesions were significantly mitigated in group pretreated with pretreatment of NKM 23-1 (*p*<0.01, Fig. 1B and C). However, the degree of rescue was more significantly noted in group pretreated with 25–50 mg/kg, though significantly attenuated, but lower in group pretreated with 100 mg/kg, denoting hormetic outcome in higher dose (Fig. 1B and C). On separate detailed analysis of pathological changes, 25–50 mg/kg NKM 23-1 significantly decreased either the mean score of gastric inflammation or erosions/ulcer index (*p*<0.05, Fig. 1C). Looking at regenerative activity, NKM 23-1 significantly increased regeneration score (*p*<0.01, Fig. 1C). Conclusively, NKM 23-1 extracts significantly prevented SRMD in a dose-dependent manner, but lesser in higher doses, possibly due to hermetic principle (Supplemental Fig. 1*). Thereafter, we put detailed molecular changes of Group 1–4 in the following experiments.

### Anti-inflammatory actions of NKM 23-1 rescued from SRMD

**Fig. 2:**
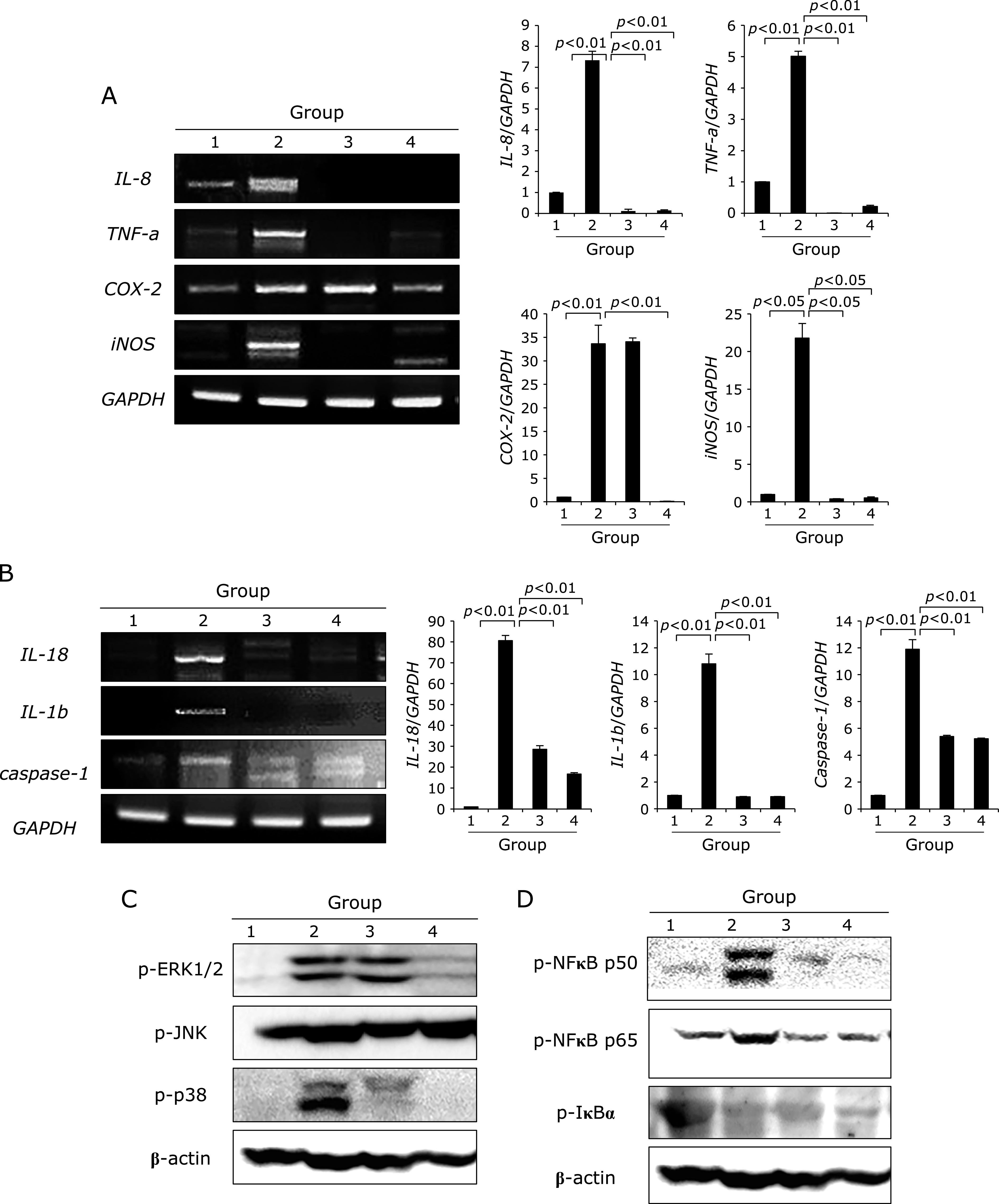
Changes of inflammatory, signal transductions, and transcription factor NF-κB according to group. (A) RT-PCR for inflammatory mediators The expressions of *IL-8*, *TNF-a*, *COX-2*, and *iNOS* mRNA were measured in all animal groups, of which expressions were all significantly increased in WIRS-induced SRMD (*p*<0.01). (B) RT-PCR for inflammasome complex RT-PCR was done for *IL-18*, *IL-1b*, and *caspase-1*. (C) Western blot for MAPKs, ERK1/2, JNK, and p38. (D) Western blot for p-NF-κB p50, p-NF-κB p65, IκBα. SRMD group showed significantly increased expressions of NF-κB p50, whereas significantly decreased IκBα.

The expressions of *IL-8*, *TNF-a*, *COX-2*, and *iNOS* mRNA were measured in all animal groups, of which expressions were all significantly increased in control group of WIRS-induced SRMD (*p*<0.001, Fig. 2A). On the other hand, these expressions of inflammatory mediators implicated in WIRS pathogenesis were all significantly decreased in group pretreated with NKM 23-1 (*p*<0.01, Fig. 2A). Under the hypothesis, NKM 23-1 might protect stomach from WIRS via the suppression of inflammasome formation, we repeated RT-PCR for IL-18, IL-1β, and capsase-3 mRNA (Fig. 2B). As expected, NKM 23-1 significantly inhibited these inflammasome components (*p*<0.001). Considering the changes of MAPKs in these inflammatory mediators, phosphorylation of ERL1/2, JNK, and p38 was measured in the stomach homogenates. As seen in Fig. 2B, phosphorylation of p38 and ERK1/2 was significantly intervened (*p*<0.001) among MAPKs. Pretreatment of NKM 23-1 significantly inactivated p38 MAPK (*p*<0.001, Fig. 2C). Checking subsequent activation of NF-κB, as seen in Fig. 2C, NF-κB p50 was significantly activated, but these activations were significantly repressed with NKM 23-1. These changes of NF-κB activation were further vilified with the changes of p-IκBα as seen in Fig. 2D. Since higher dose of NKM 23-1, 100 mg/kg, showed inferior efficacy compared to either 25 or 50 mg/kg NKM 23-1 (Supplemental Fig. 1*), we added the measurement of MAPKs and NF-κB including Group 5, as seen in Supplemental Fig. 2A and B*, NKM 23-1 led to rescuing action against WIRS via inactivation of ERK1/2/ p38 or NF-κB, but dose lesser than 100 mg/kg.

### Increased infiltrations of macrophages with WIRS were significantly decreased with NKM 23-1

**Fig. 3:**
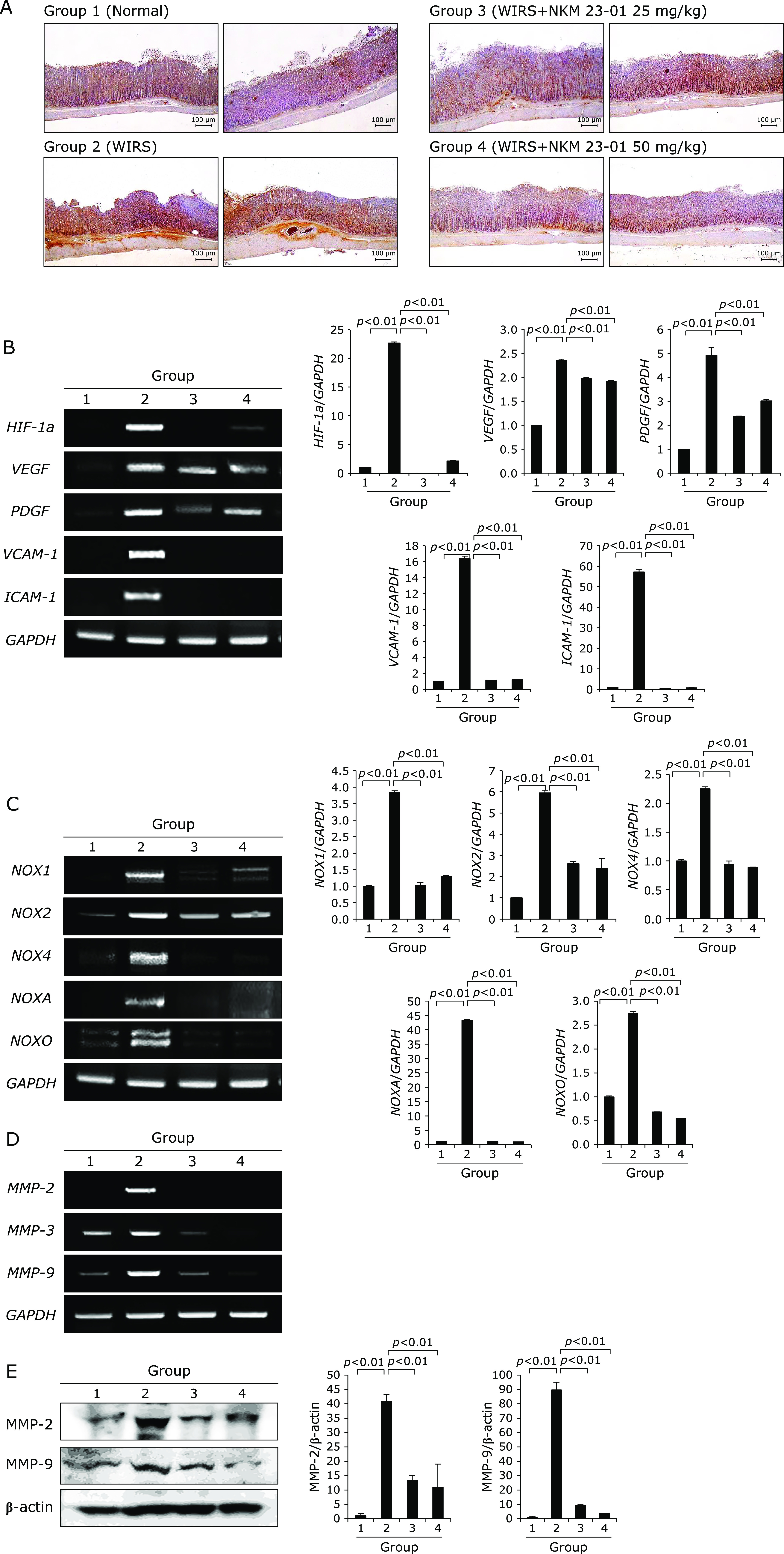
Macrophages and associated mediators according to group. (A) Immunohistochemical stainings for macrophages. Significantly increased levels of F4/80 positive immunostainings were noted in Group 2 (*p*<0.001), whereas F4/80 positive stainings were significantly decreased in group pretreated with NKM 23-1. ×40 magnification. (B) RT-PCR for angiogenic growth factors such as *HIF-1a*, *VEGF*, *PGDF* and adhesion molecules including *VCAM-1* and *ICAM-1*. Angiogenic growth factors, including *HIF-1a*, *PDGF*, and *VEGF* mRNA were measured. The expressions of *HIF-1a*, *PDGF*, and *VEGF* were all significantly increased in SRMD group (*p*<0.01), while these expressions were all significantly decreased in either Group 3 or Group 4 (*p*<0.01). (C) RT-PCR for NADPH oxidases (NOXs) (D, E) RT-PCR for matrix metalloproteinases (MMPs) and Western blots for MMP-2 and MMP-9.

F4/80 positive macrophages were significantly increased after WIRS, leading to the above changes of inflammatory mediators (Fig. 3A). However, pretreatment of NKM 23-1 significantly decreased the expressions of F4/80 in the stomach, signifying the inhibition of macrophage infiltrations relevant to WIRS (*p*<0.001, Fig. 3A). These macrophage infiltrations led to significant expressions of inflammasome, as seen in Fig. 2B, *IL-18*, *IL-1b*, and *caspase-1* mRNA. Furthermore, the expressions of *HIF-1a*, *VEGF*, *PDGF*, *VCAM-1*, and *ICAM-1* mRNA were all significantly increased in WIRS control group (Fig. 3B), signifying that pathogenesis of WIRS was related to ischemia and defective blood supply. However, these expressions of hypoxia and angiogenic growth factors were all significantly ameliorated in NKM 23-1 pretreatment (Fig. 3C). We further measured the changes of NADPH oxidase (NOX) according to group. As seen in Fig. 3C, expressions of *NOX1*, *NOX2*, *NOX3*, *NOX4*, *NOXA*, and *NOXO* mRNA were all significantly increased with WIRS, whereas NKM 23-1 was significantly decreased these expressions of *NOX*s mRNA (*p*<0.01). We extended our measurement of matrix metalloproteinases (MMPs) and as seen in Fig. 3D and E, the expressions of MMPs were significantly increased after WIRS, but pretreatment of NKM 23-1 all significantly decreased the expressions of MMP-2 and MMP-9 (*p*<0.01).

### Anti-apoptotic actions of NKM 23-1 led to mitigating action against SRMD

**Fig. 4:**
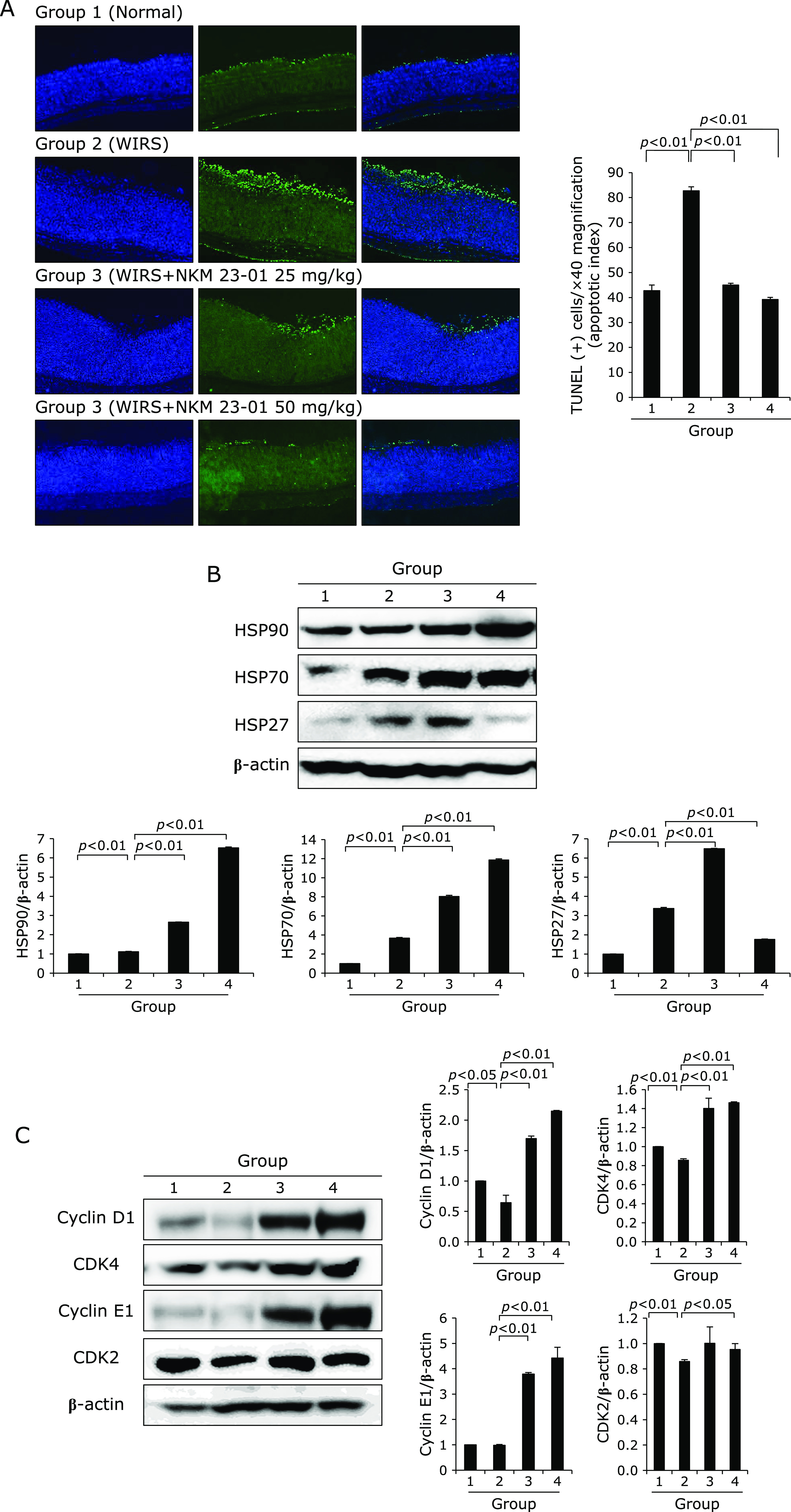
Changes of apoptosis, heat shock proteins, and cell cycle protein according to group. (A) TUNEL for apoptotic cells SRMD as seen in Group 2 was associated with increased apoptotic event in eroded or ulcerative SRMD area. Apoptotic index was significantly increased in SRMD group, but NKM 23-1 pretreatment significantly decreased apoptosis even in erosive gastric mucosa (*p*<0.01), ×40 magnification. (B) Western blot for heat shock proteins (HSPs). In order to confirm the findings from TUNEL, Western blots for anti-apoptotic chaperone proteins, HSPs, were done. SRMD alone increased the expressions of HSP70 and HSP27 as compensatory host response against apoptotic challenges. However, the expressions of HSP70 and HSP27 were significantly increased with NKM 23-1 pretreatment (*p*<0.001). (C) Western blot for cyclins and CDKs. The expression of CDK4 and cyclin D1 were significantly increased in SRMD control groups (*p*<0.05), whereas the expressions of CDK4 and cyclin D1 were significantly decreased in group pretreated with NKM 23-1 pretreatment (*p*<0.01).

SRMD is known to occur with robust apoptosis.^(17)^ As seen in Fig. 4A, apoptotic index was significantly increased in SRMD group (*p*<0.01). Instead, Pretreatment of NKM 23-1 significantly decreased apoptosis even in erosive gastric mucosa (*p*<0.01). Besides of anti-apoptotic privilege of NKM 23-1 as seen in Fig. 4A, the expressions of molecular chaperone protein, heat shock proteins (HSPs), were significantly preserved and increased with NKM 23-1. As seen in Fig. 4B, significant inductions of HSP 70 were noted with NKM 23-1 (*p*<0.05, Fig. 4B). Simultaneously, measuring the changes of HSPs, as can be seen in Fig. 4B, though significantly increased expressions of HSP70 and HSP27 as part of molecular chaperone, more significant inductions of HSP70 and HSP27 were noted with the pretreatment of NKM 23-1 (*p*<0.01, Fig. 4B). Resistant to apoptotic induction after SRMD, regeneration can be supported with the induction of cell cycle, as seen in Fig. 4C, the expressions of cyclin D1, cyclin E1, CDK4 were significantly increased with pretreatment of NKM 23-1 (*p*<0.01, Fig. 4C).

### Operation of host phase 2 defensive mechanisms with NKM 23-1 in WIRS model

**Fig. 5:**
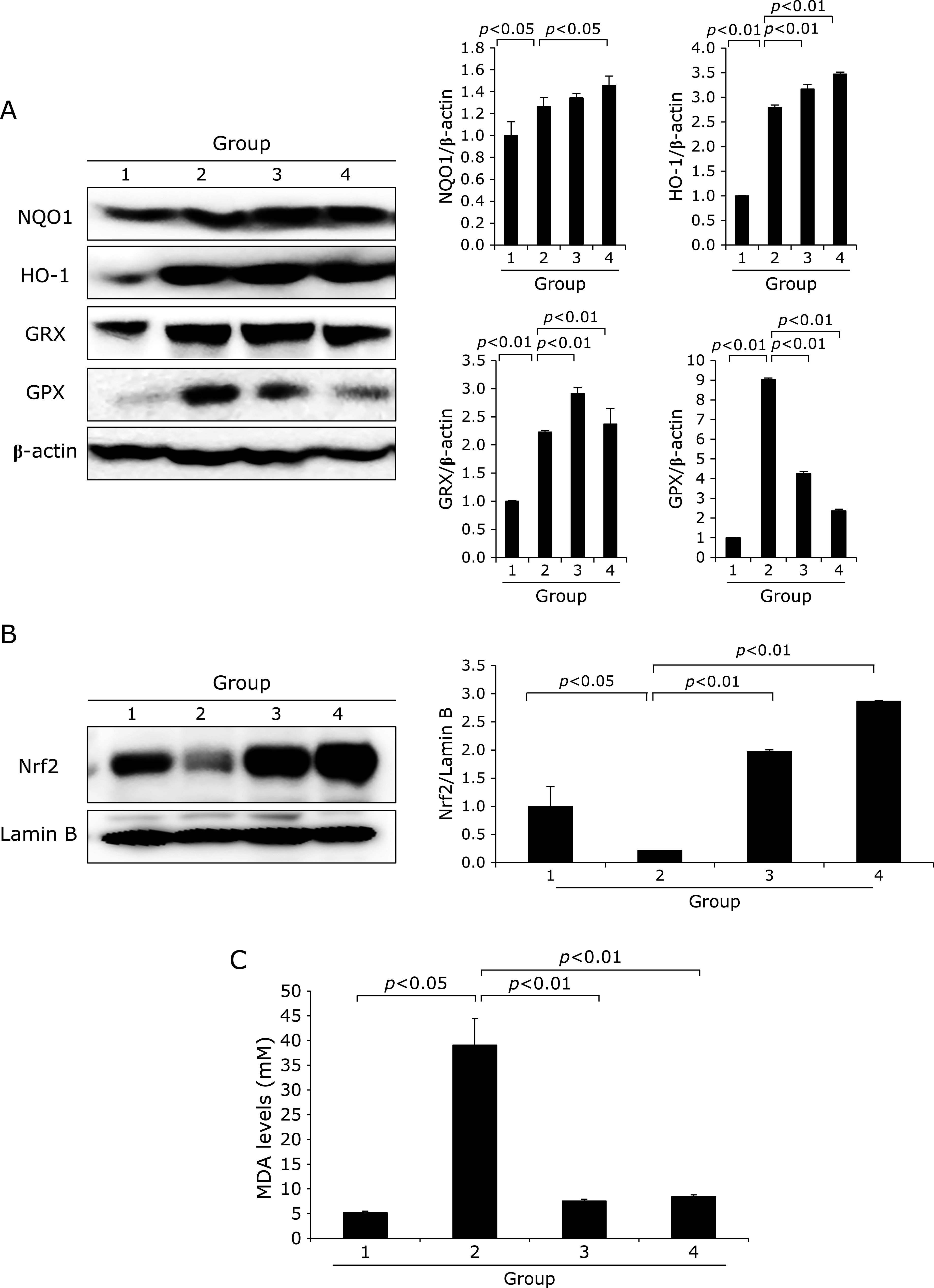
Changes of host defensive factors according to group. (A)Western blot for NQO1, HO-1, GRX, and GPX according to group. WIRS induced phase 2 host defensive proteins as innate defense after WIRS, but the expressions of NQO1 and HO-1 were significantly increased in group pretreated with NKM 23-1 (*p*<0.05) NQO1 denotes NAD(P)H dehydrogenase (quinone) 1, HO-1 denoted hemeoxygenase-1, GRX denoted glutaredoxin, and GPX denoted glutathione peroxidase. (B) Western blot for Nrf2. Using nuclear homogenates in each group, the Western blot for transcription factor Nrf2 showed significant ablation in WIRS control group, whereas Nrf nuclear translocation was significantly increased in group pretreated with NKM 23-1. (C) ELISA levels of MDA reflecting lipid peroxidation.

Under the hypothesis that pretreatment of NKM 23-1 can increase host cytoprotective proteins such as HO-1, NQO1, and GPX, as noted in Fig. 5A, NKM 23-1 significantly induced HO-1 and NQO1 (*p*<0.05). Therefore, we measured the transcription factor for HO-1, Nrf2 in nuclear homogenates. As seen in Fig. 5B, nuclear translocated expressions of Nrf2 were significantly increased in group pretreated with NKM 23-1 (*p*<0.001). Interestingly, WIRS alone increased host defense system presented with some induction of HO-1 and NQO1 (*p*<0.05, Fig. 5A and B), but its expressions were further significantly increased in group pretreated with NKM 23-1 (*p*<0.01, Fig. 5), inferring that WIRS-induced oxidative stress significantly led to HO-1 expression in control rats as part of host defense,^(18,19)^ but its levels were further significantly increased in group pretreated with NKM 23-1, strengthening cytoprotection via phase 2 enzyme response under SRMD.

### Significant inhibition of WIRS-related lipid peroxidation

WIRS model reflected significant ischemia/reperfusion condition via stress, after which oxidative stress prevails in the affecting stomach. When MDA levels were measured according to group, as expected, the levels of MDA were significantly increased in the stomach homogenates, but pretreatment of NKM 23-1 significantly decreased lipid peroxidation (*p*<0.01, Fig. 5C).

### Preserved gastric mucin with NKM 23-1 pretreatment rescued from SRMD

**Fig. 6:**
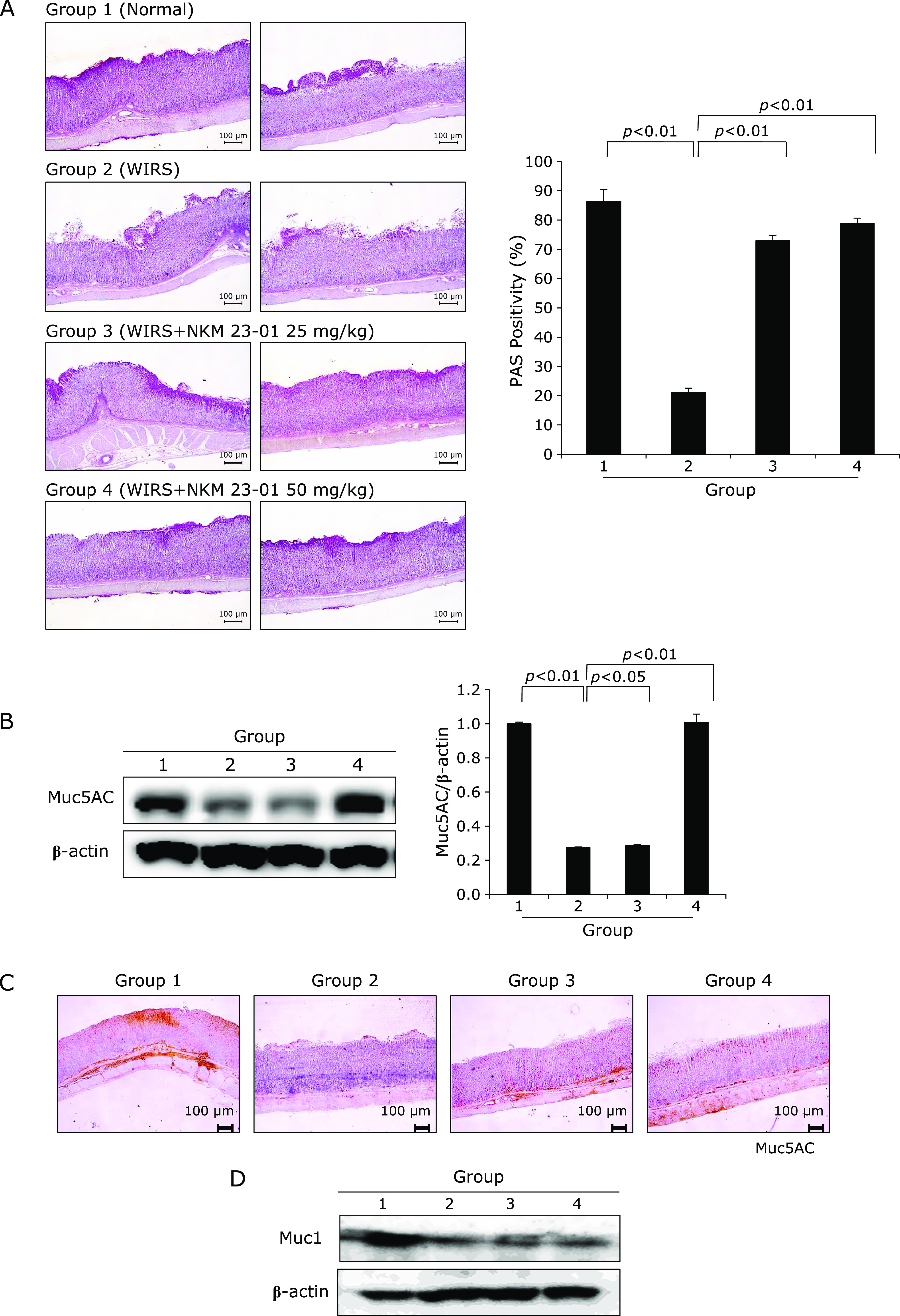
Changes of gastric mucus according to group. (A) PAS staining for gastric mucin. Significantly decreased levels of gastric mucin were noted in Group 2 (*p*<0.01), whereas the expressions of gastric mucin were significantly preserved in group pretreated with NKM 23-1 (*p*<0.01), ×40 magnification of PAS staining. (B) Western blot for Muc5A. (C) Immunohistochemical staining for Muc5A (D) Western blot for Muc1 WIRS led to significant decreases in Muc1 (*p*<0.05), but slight increment with NKM 23-1 pretreatment without statistical significance.

Gastric mucin and mucus layers imposed significant pre-epithelial and epithelial defense system in the stomach,^(10)^ in which mucus layer plays major contribution to cytoprotection. However, as seen in Fig. 6A, WIRS led to significant depletion of covering mucus layer due to insufficient blood supply, deranged synthesis, and increased degradation. Under the hypothesis that NKM 23-1 might contribute to mucus preservation, we measured the distribution of gastric mucus layer with PAS staining. As result, WIRS significantly depleted gastric mucus (*p*<0.001), whereas the mucus layer was significantly preserved in spite of applying WIRS (Fig. 6A). When Muc5A expression was measured according to group, significantly preservation of Muc5A was noted in NKM 23-1 pretreatment (Fig. 6B). In addition, we have added the changes of Muc5A immunohistochemical staining, as seen in Fig. 6C, similar preservation of Muc5A was noted in NKM 23-1 pretreated group (*p*<0.05, Fig. 6C). The expressions of Muc1 was significantly decreased after WIRS (*p*<0.05), but slightly preserved with NKM 23-1 without statistical significance (Fig. 6D).

## Discussion

**Fig. 7:**
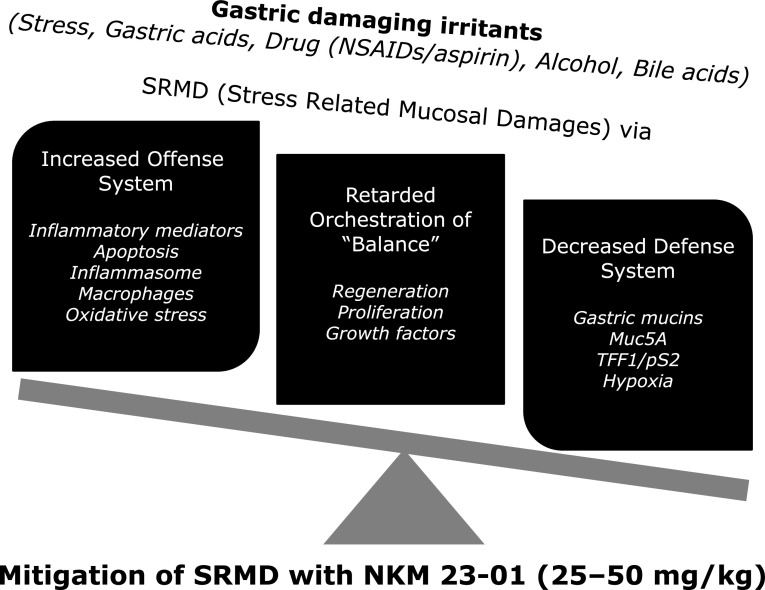
Schematic summary how NKM 23-1 afforded rescuing action against WIRS. Balance between offensive factors and defensive factors is quite essential in the stomach. Therefore, as increasing unmet medical needs for SRMD, preemptive administration of reliable nutrient or agent is prerequisite. In this condition, as summarized in schematic figure, current study clearly showed the administration of NKM 23-1 significantly corrected pathogenic mechanisms of SRMS, for instance, decreasing offense system such as inflammatory mediators, apoptosis, inflammasome, macrophage infiltration, oxidative stress, increasing defense system such as gastric protective mucins, Muc5A, relieving hypoxia, and balancing of regeneration, proliferation, and growth factors under concerted manner, reaching to the conclusion that NKM 23-1 (*Dolichos lablab* L. extracted from white bean) can be pharmanutrient to mitigate SRMD.

In the current study, we reached to the conclusion that NKM 23-1 (*Dolichos lablab* L. extracted from white bean) can be “pharmanutrient” to protect from SRMD since either the removal of irritants or only ulcer prophylaxis is reliable way of treatment. As increasing unmet medical needs, preemptive administration of reliable nutrient or agent is prerequisite. In this condition, as summarized in Fig. 7, our study clearly showed the administration of NKM 23-1 significantly corrected pathogenic mechanisms of SRMS, decreasing offense system (including inflammatory mediators, apoptosis, inflammasome, macrophage infiltration, and oxidative stress), increasing defense system (gastric protective mucins, Muc5A, and relieving hypoxia), balancing of regeneration, proliferation, and growth factors under concerted manner. Different with gastric cytoprotective drugs such as rebamipide, ecabet sodium, and teprenon, these cytoprotective nutrients follow up the principle of hormesis, that is, too much is not always better.

As seen in current experiment, though NKM 23-1 exerted significant cytoprotection against SRMD, interestingly, 25 to 50 mg/kg showed higher efficacy than 100 mg/kg. In other models of gastric ulcer, ethanol-induced or HCl-induced, same results were noted. Some molecular data also supported these unexpected outcomes. However, significant cytoprotection efficacy of NKM 23-1 might be based with the concept of “hormesis”, concept initiated at toxicology and microbiology, but well applied in dietary regimen as well as medicine,^(20)^ in detail, hormesis enhances resilience to normal aging, protects against neurodegenerative, cardiovascular, and other destructive trauma, other threats to digestive tract damages including gastric ulcer, and affords adaptive protections.^(21)^ Focusing on concept of hormesis relevant to cytoprotection, hormesis has emerged a central concept in biomedical sciences that too much is not always better.^(22)^ However, hormetic events should be under “lower-dose stress” within spectrum apoptosis or autophagy in cell biology.^(23)^ As meant by ‘What doesn’t kill you makes you stronger, hormesis, the paradoxical beneficial effects of stressors,’ can be better defined as the biphasic dose-effect. As observed in our experiment (Fig. 1), an initial exposure can elicit an efficient adaptive stress response with long-lasting protection against WIRS, while as seen in Supplementary Fig. 1, 100 mg/kg NKM 23-1 failed in imposing protection from WIRS. As evident in MAPKs assay and NF-κB repression (Supplemental Fig. 2*), Group 5 showed different ERK1/2 inactivation and NF-κB repression of Group 3 and Group 4. Though not shown in results, these hormetic efficacies of NKM 23-1 were also noted in model with HCl- and ethanol-stimulated gastric damages.

Though the term of SRMD is often confusing, with “*stress ulcer*”, “*stress erosions*”, and “*stress lesions*”,^(2–4)^ ultimate pathogenesis shared same pathogenesis of small superficial erosions to overt gastric ulcers, but all related to “stress response” in the stomach.^(24)^ In non-stress condition, mucosal defense consisted of adequate microcirculation, efficient regenerating capability, and neurogenic protective innervation, but simultaneously removals of waste products, free radicals scavenging, wasting secretion and any irritants also are operating. However, in stress condition, inefficient perfusion rather increased gastric acidity, leading to formation of gastric lesions.^(25)^ Therefore, in clinic, ulcer prophylaxis and adequate care for circulation are core therapeutic approach, thereafter, removal of mucosal irritants such as gastric acid and agent capable of enhancing gastric defense systems and securing perfusion are main stay of treatment for SRMD. However, still improvement as unmet medical needs is prerequisite because ulcer prophylaxis with anti-secretary therapy can only reduce bleeding, never can reduce mortality and prevent gastric pathology. With the results from lignin, Kwiecien *et al.*^(26)^ proved the efficacy of pentoxifylline, an inhibitor of TNF-α activity against WIRS-induced gastric lesions, the efficacy of olive leaf extract,^(27)^ and prickly pear cactus,^(28)^ in which commonly significant scavenging action of free radicals was noted.^(29)^ Among several searches for ideal agent to mitigate SRMD, we reached to apply NKM 23-1 based on preliminary exploration that NKM 23-1 significantly reduced the severity of HCl-induced gastric ulcer and ethanol-associated damages (data not shown).

Mucin is biosynthesized within mucus-producing cells and secreted from them, largely two kinds of cells including surface mucus cells and gland mucus cells and core peptides are characterized as MUC5AC and MUC6, respectively, in which amino acids like threonine and serine are used for forming core peptide.^(30)^ Following biosynthesis in mucus-producing cells, mucin accumulates as mucus granules in the cells and is subsequently secreted through exocytosis.^(31,32)^ In this study, PAS positive mucus cells were significantly decreased after WIRS, but significant preservation of PAS positive mucus cells were noted in Group 3 and Group 4, pretreated with NKM 23-1, signifying preserving mucin with NKM 23-1 pretreatment afforded significant protection against SRMD.

In the current investigation, significant cytoprotection of NKM 23-1 was achieved through Nrf2-mediated HO-1 induction. Similar actions documenting Nrf2 mediated protection with natural extracts were explored in several models. In addition to our group already revealing that extract from *Artemisia* attenuated SRMD via Nrf2-mediated mucoprotective and anti-inflammatory action,^(33)^ Dinkova-Kostova *et al.*^(34)^ shown beneficial effect of sulforaphane targeting Nrf2 and Song *et al.*^(35)^ published dithiolethione extracts exerted neuroprotection via activated Nrf2-driven antioxidant enzyme. Since keap1-Nrf2 (*Kelch*-like ECH-associated protein 1-NF-E2-related factor 2) system, keap1 as a cysteine thiol-rich sensor and Nrf2 as transcription factor regulating a battery of cytoprotective genes, HO-1, NQO1, γ-GCL etc, forms the major node of cellular defense against stress, controlling the keap1-Nrf2 as a key to healthy condition and well-being.^(36)^ In our experiment, we could document these Nrf2 systems operated in accordance with regulation of inflammasomes (Fig. 2B). Inflammasomes represent central regulators of inflammation. Upon detection of various stress factors, assembly of the inflammasome protein complex results in activation and secretion of proinflammatory cytokines because inflammasomes are central regulator of inflammation.^(37)^ Since growing evidences of a crosstalk between the Nrf2 and inflammasome pathways has accumulated, Nrf2 activation with NKM 23-1 significantly inhibited inflammasomes and consequently inflammation. HO-1 transcribed via Nrf2 is a stress-inducible enzyme that has been shown to confer significant protection in the stomach as well as various organ system.^(38)^ Cytoprotective, anti-inflammatory, antioxidant, and anti-apoptotic activities of HO-1 provide secure therapeutic target for WIRS.

In addition, HSPs have been implicated in gastric defense mechanisms at the intracellular level. Certain HSPs are expressed under non-stressful conditions and play an important role in the maintenance of normal cell integrity by virtue of chaperon function, but HSPs are also considered to improve cellular recovery both by either refolding partially damaged functional proteins or increasing delivery of precursor proteins to important organelles such as mitochondria and endoplasmic reticulum.^(39)^ Conclusively, in this investigation, authors were very successful in documenting the extracts of *Dolichos lablab* L. as candidate to alleviate SRMD to the level of “pharmanutrient”. The term “pharmanutrient” is not popular term, but our authors proudly stated that “the link between diet and human health” and “diet and drug as same origin” has been proved by several scientific evidences and functional foods, food supplements,^(40)^ food extracts, and nutraceuticals are at the interface between nutrition and drug. Though the concept of ‘cytoprotection’ and adaptive cytoprotection had been developed to describe the ability of pharmacologic agents, for instance, prostaglandins, to reduce damage to the stomach induced by gastric irritants, current study consistently documented NKM 23-1 can be of potential candidate as “pharmanutrient” to improve troublesome SRMD after well-designed RCT in a near future.

## Author Contributions

Study concept and design: DJS and KBH; acquisition of data: JMA, EHK, and HJL; analysis and statistical analysis: JMA, EHK, HJL; interpretation of data: JMA, DJS, MHP; drafting of manuscript: HJL and KBH

## Figures and Tables

**Table 1:** Primers used in RT-PCR

Gene	Forward primer (5'→3')	Reverse primer (5'→3')
IL-8	CACTCCCAGCATCGTAGAGC	CAGTGTACTTGTGGCGTGGA
iNOS	TTTTCCCAGGCAACCAGACG	GTAGCGGGGTTCAGAATGG
TNF-α	CCCTCACACTCAGATCATCTTCTCAA	TCTAAGGTACTTGGGCAGGTTGACCTC
IFN-γ	ATCCATGAGTGCTACACGCC	TCTGTGGGTTGTTCACCTCG
IL-6	CTTCCAGCCAGTTGCCTTCT	GAGAGCATTGGAAGTTGGGG
HIF-1α	TATCACTGGACTTCGGCAGC	GCTGCCGAAGTCCAGTGATA
PDGF	AGGAAGCCATTCCCGCAGTT	CTAACCTCACCTGGACCTCT
VEGF	CAATGATGAAGCCCTGGAGT	GATTTCTTGCGCTTTCGTTT
GAPDH	GGTGCTGAGTATGTCGTGGA	TTCAGCTCTGGGATGACCTT

## Abbreviations

HO-1: heme oxygenase-1
HSP: heat shock protein
ICU: intensive care unit
MMP: matrix metalloproteinase
NKM 23-1: *Dolichos lablab* L. extract
SRMD: stress-related mucosal disease
WIRS: water immersion restraint stress
