# Supplementary material for: Dolichos lablab L. extracts as pharmanutrient for stress-related mucosal disease in rat stomach
Source: J Clin Biochem Nutr. 2020 Jun 11;67(1):89–101. doi: 10.3164/jcbn.20-11 (PMC7417803; doi:10.3164/jcbn.20-11)
Supplement: Supplemental Figure 1 [file jcbn20-11sf01.pdf]

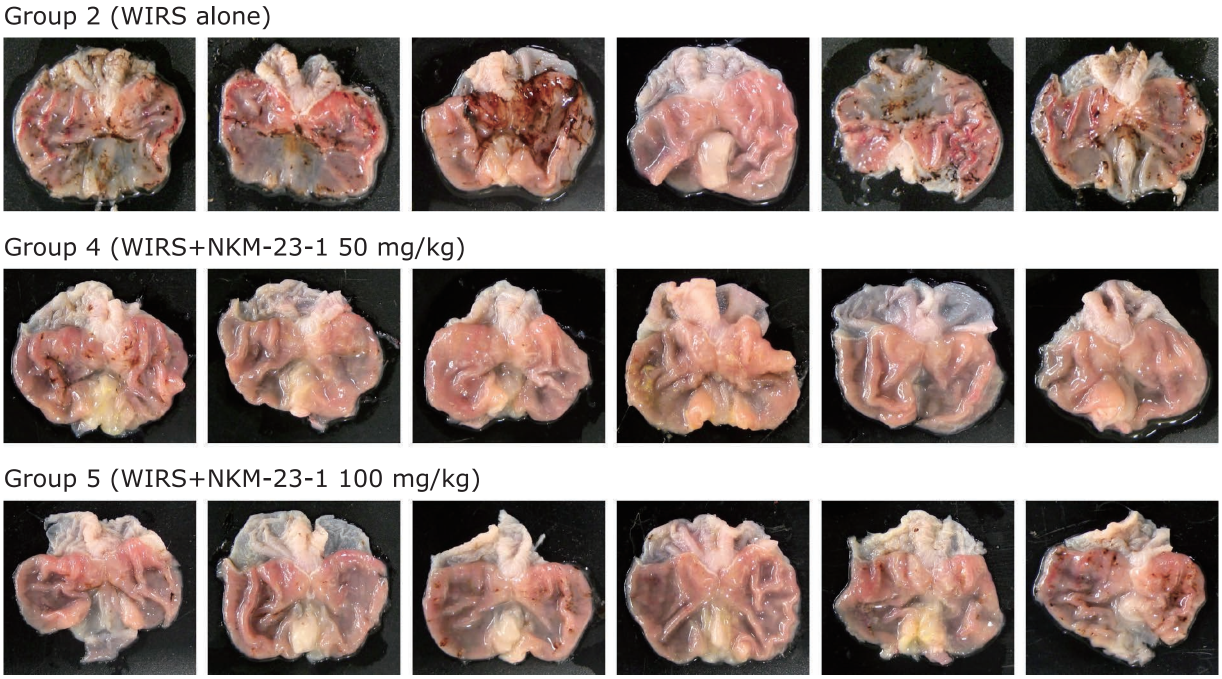

Supplemental Fig. 1. Gross morphology according to group Compared to control (WIRS group), Group 5 pretreated with 100 mg/kg NKM 23-1 showed lesser protection from WIRS than Group 4 pretreated with 50 mg/kg NKM 23-1. Hormetic effect that higher dose showed lesser efficacy than lower dose was noted with 100 mg/kg NKM 23-1.
