# Supplementary material for: Dolichos lablab L. extracts as pharmanutrient for stress-related mucosal disease in rat stomach
Source: J Clin Biochem Nutr. 2020 Jun 11;67(1):89–101. doi: 10.3164/jcbn.20-11 (PMC7417803; doi:10.3164/jcbn.20-11)
Supplement: Supplemental Figure 2 [file jcbn20-11sf02.pdf]

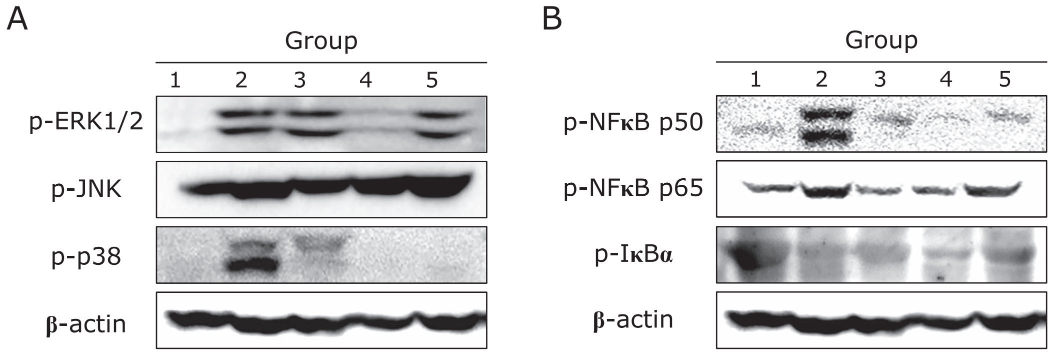

Supplemental Fig. 2. Measurement of MAPKs and NF-κB in Group 5 NKM 23-1 showed rescuing action from WIRS through either inactivation of p38 or NF-κB repression in Group 3 or Group 4, but these signaling were different in Group 5 (A) MAPKs (B) NF-κB.
